# Disease-stabilizing treatment based on all-trans retinoic acid and valproic acid in acute myeloid leukemia – identification of responders by gene expression profiling of pretreatment leukemic cells

**DOI:** 10.1186/s12885-017-3620-y

**Published:** 2017-09-06

**Authors:** Håkon Reikvam, Randi Hovland, Rakel Brendsdal Forthun, Sigrid Erdal, Bjørn Tore Gjertsen, Hanne Fredly, Øystein Bruserud

**Affiliations:** 1 Department of Medicine, Haukeland University Hospital, N-5021 Bergen, Norway; 2 Center for Medical Genetics and Molecular Medicine, Haukeland University Hospital, Bergen, Norway; 3 Section for Hematology, Institute of Clinical Science, University of Bergen, Bergen, Norway

**Keywords:** Acute myeloid leukemia, All-trans retinoic acid, Valproic acid, Gene expression profiling

## Abstract

**Background:**

Acute myeloid leukemia (AML) is an aggressive malignancy only cured by intensive therapy. However, many elderly and unfit patients cannot receive such treatment due to an unacceptable risk of treatment-related morbidity and mortality. Disease-stabilizing therapy is then the only possible strategy, one alternative being treatment based on all-trans retinoic acid (ATRA) combined with the histone deacetylase inhibitor valproic acid and possibly low-toxicity conventional chemotherapy.

**Methods:**

Primary AML cells were derived from 43 patients included in two clinical studies of treatment based on ATRA, valproic acid and theophyllamine; low toxicity chemotherapy (low-dose cytarabine, hydroxyurea, 6-mercaptopurin) was also allowed. Pretreatment leukemic cells were analyzed by mutation profiling of 54 genes frequently mutated in myeloid malignancies and by global gene expression profiling before and during in vivo treatment.

**Results:**

Patients were classified as responders and non-responders to the treatment, however response to treatment showed no significant associations with karyotype or mutational profiles. Significance analysis of microarray (SAM) showed that responders and non-responders significantly differed with regard to the expression of 179 different genes. The differentially expressed genes encoding proteins with a known function were further classified based on the PANTHER (protein annotation through evolutionary relationship) classification system. The identified genes encoded proteins that are involved in several important biological functions, but a main subset of the genes were important for transcriptional regulation. These pretherapy differences in gene expression were largely maintained during treatment. Our analyses of primary AML cells during in vivo treatment suggest that ATRA modulates HOX activity (i.e. decreased expression of *HOXA3*, *HOXA4* and *HOXA*5 and their regulator *PBX3*), but altered function of DNA methyl transferase 3A (DNMT3A) and G-protein coupled receptor signaling may also contribute to the effect of the overall treatment.

**Conclusions:**

Responders and non-responders to AML stabilizing treatment based on ATRA and valproic acid differ in the pretreatment transcriptional regulation of their leukemic cells, and these differences may be important for the clinical effect of this treatment.

**Trial registrations:**

ClinicalTrials.gov no. NCT00175812; EudraCT no. 2004–001663-22, registered September 9, 2005 and ClinicalTrials.gov no. NCT00995332; EudraCT no. 2007–2007–001995-36, registered October 14, 2009.

**Electronic supplementary material:**

The online version of this article (10.1186/s12885-017-3620-y) contains supplementary material, which is available to authorized users.

## Background

Acute myelogenous leukemia (AML) is an aggressive malignant disease of the bone marrow in which hematopoietic precursors are arrested in an early stage of development. AML are distinguished from other related blood disorders by the presence of >20% blasts in the bone marrow [1]. The only possibility for cure is intensive induction chemotherapy followed by consolidation treatment with intensive chemotherapy or stem cell transplantation, although for various reasons this treatment is not possible for several elderly or unfit patients [2–4]. Firstly, elderly patients have a higher and often unacceptable risk of severe treatment-related complications compared with younger patients [2–4]. The median age at the time of diagnosis of AML is 65–70 years and elderly patients thus represent the largest group of AML patients [1]. Secondly, unfit patients with severe comorbidity also have an unacceptable risk of severe complications and treatment-related mortality. Thirdly, several patients with relapsed or resistant disease will not receive further intensive treatment [5]. All these groups constitute a relatively large patient population that should be considered for AML-stabilizing treatment, e.g. treatment based on all-trans retinoic acid (ATRA) + valproic acid and low-toxicity cytotoxic treatment with hydroxyurea, 6-mercaptopurin or low-dose cytarabine [6–11].

New treatment approaches are currently considered for AML patients unfit for intensive chemotherapy. A promising concept is modulation of protein lysine acetylation through inhibition of histone deacetylases (HDACs) [12]. These enzymes alter acetylation of histones as well as transcription factors and other proteins involved in the regulation of cellular proliferation and survival. Valproic acid has features as a HDAC inhibitor, and are currently investigated in clinical studies of elderly or unfit AML patients, often in combination with ATRA [13, 14]. The toxicity of this treatment is low. Complete hematological remission lasting for several months has been reported for a minority (<5–10%) of patients but increased peripheral blood platelet counts are seen for 30–40% of patients and may last for up to 1–2 years [13, 14]. Valproic acid and ATRA may also be combined with conventional low-toxicity chemotherapy [7, 13, 14].

ATRA is a vitamin A metabolite that binds to retinoid-responsive nuclear receptors and thereby exerts effects on cell growth, differentiation and apoptosis [15]. It is used in the treatment of acute promyelocytic leukemia (APL) [16], although may also have antileukemic effects in non-APL variants of AML [17–19]. HDAC-inhibitors can reduce proliferation and induce differentiation in malignant hematopoietic cells [20], and these effects seem to be enhanced in combination with ATRA [21, 22]. The combination of valproic acid, ATRA and possibly low-toxicity chemotherapy has been examined in several clinical studies of AML patients with non-APL disease [14]. In this context we compared genetic abnormalities and gene expression patterns for responders and non-responder patients to low-toxicity treatment based on the combination of ATRA and valproic acid.

## Methods

### Patient characterization and classification

#### Patients included

A large group of consecutive AML patients unfit for intensive chemotherapy was included in two different phase 1/2 studies [9, 13]. Both the first study, including 24 patients, and the second study, including 36 patients were approved, by the Regional Ethics committee (REK Vest 215.03 and 231.06, respectively) and registered in a public database (for the first study ClinicalTrials.gov number NCT00175812 and EudraCT number 2004–001663-22; for the second study ClinicalTrials.gov number NCT00995332 and EudraCT number 2007–2007–001995-36 respectively). All patients were included after written informed consent.

A total of 60 patients unfit for more intensive therapy were included in the two studies; their characteristics are summarized in Additional file 1: Table S1. A majority of them were elderly patients with high-risk disease (i.e. leukemia relapse, secondary AML or high-risk cytogenetic abnormalities). Detailed information of all patients included in our present study are given in Additional file 1: Table S2.

During the time periods for the two clinical studies 9 additional patients unfit for intensive treatment were also diagnosed at our department; these patients were not included in the clinical studies because they (i) did not accept inclusion (1 patient); (ii) informed consent (6 patients) or (iii) adequate follow-up was not possible (1 patient); and (iv) hydroxyurea treatment was already started (1 patient).

The antileukemic treatments in the two clinical studies are summarized in Additional file 1: Table S3. Both protocols were based on intermittent ATRA therapy for 2 weeks at 12 weeks intervals, continuous oral valproic acid treatment and additional low-dose chemotherapy given either as (i) hydroxyurea (daily)/6-mercaptopurine (daily)/low-dose cytarabine (at least 4 weeks intervals) to maintain peripheral blood blast counts below 50 × 10^9^/L (the 24 patient in study 1) [9]; or (ii) low-dose cytarabine at 12 weeks intervals as long as peripheral blood blast counts were below 50 × 10^9^/L, this being replaced by oral hydroxyurea/6-mercaptopurin if blast counts increased (the 36 patients in study 2) [13]. A total of 28 patients were included in the global gene expression studies of pretreatment primary AML cells (see Table 1; 17 from the first and 11 from the second study; 14 males and 14 females; median age 76 years with range 48–87 years). Only 16 of the 28 patients had de novo AML, the others had either AML secondary to chronic myeloproliferative neoplasia (3 patients), previous chemotherapy (1 patient) or myelodysplastic syndrome (MDS, 5 patients); 3 additional patients had AML relapse. Seventeen patients had normal karyotype and 7 had adverse karyotype; 10 patients had FLT3-ITD. Twenty-three patients could be classified as having unfavorable prognosis having at least one of the following criteria: High-risk karyotype (7 patients), AML relapse (3 patients) or secondary AML (9 patients).

**Table 1:** Comparison of global gene expression profiles for responders and non-responders to AML stabilizing treatment based on ATRA and valproic acid – a summary of the results from the Gene Set Enrichment Analysis (GSEA)

GENE SET	SIZE	ES	NES	NOM *P*-VALUE
Signal sequence binding	21	−0.62	−1.85	<0.01
Olfactory bulb interneuron development	10	−0.72	−1.77	<0.01
Negative regulation of MAPK cascade	38	−0.5	−1.76	<0.01
Response to ionizing radiation	85	−0.45	−1.69	<0.01
Zinc ion transport	16	−0.67	−1.76	0.01
Pre-mRNA binding	13	−0.7	−1.73	0.01
Positive regulation of oxidoreductase activity	14	−0.65	−1.73	0.01
Inactivation of MAPK activity	27	−0.48	−1.62	0.01
Regulation of interferon-gamma biosynthetic process	16	−0.6	−1.61	0.01
Carboxylic acid catabolic process	34	−0.45	−1.61	0.01
Response to radiation	189	−0.37	−1.56	0.01
Negative regulation of reactive oxygen species metabolic process	11	−0.64	−1.68	0.02
Regulation of bone resorption	10	−0.7	−1.66	0.02
RNA polymerase II transcription factor binding transcription factor activity involved in negative regulation of transcription	11	−0.62	−1.65	0.02
Biotin metabolic process	10	−0.73	−1.65	0.02
Regulation of isotype switching	20	−0.55	−1.64	0.02
Fucosyltransferase activity	14	−0.64	−1.65	0.03
Microtubule bundle formation	24	−0.52	−1.6	0.03
Negative regulation of JNK cascade	22	−0.59	−1.59	0.03
UV protection	11	−0.6	−1.56	0.03
MutSalpha complex binding	11	−0.72	−1.59	0.04
Aminopeptidase activity	34	−0.47	−1.58	0.04
Mismatch repair complex	10	−0.74	−1.57	0.04
Negative regulation of transcription by competitive promoter binding	10	−0.61	−1.56	0.04
Cholesterol efflux	25	−0.51	−1.55	0.04
Negative regulation of cytoskeleton organization	51	−0.41	−1.51	0.04
Regulation of protein localization to cell surface	17	−0.53	−1.51	0.04
Response to gamma radiation	32	−0.44	−1.47	0.04

*Abbreviations*: *ES* enrichment score *JNK* c-Jun N-terminal kinases, *MAPK* mitogen-activated protein kinase, *NES* normalized enrichment score

#### Treatment

Patients included in the Study 1 were treated with oral ATRA 22.5 mg/m^2^ twice daily days 1–14, and valproic acid together with theophyllamine from day 3 until disease progression [9]. The treatment with valproic acid and theophyllamine started with an initial intravenous loading dose followed by 48 h of intravenous infusion guided by the serum levels before the oral treatment. For valproic acid the loading dose of 5 mg/kg was administered during 30 min and continued as an intravenous infusion of 28 mg/kg/24 h. For theophyllamine the loading dose of 5 mg/kg was administered over 30 min and continued as an intravenous infusion of 0.65 mg/kg/h. Samples were collected before treatment (day 1), after 2 days of treatment with ATRA alone (day 3) and after 5 additional days of treatment with the triple combination (day 8). ATRA was repeated with 12 weeks intervals (Study registration: ClinicalTrials.gov no. NCT00175812 and EudraCT no.2004–001663-22).

Patients included in study 2 were treated with valproic acid from day 1 and until disease progression, oral ATRA 22.5 mg/m^2^ twice daily days 8–22 and subcutaneous cytarabine 10 mg/m^2^ administered once daily on days 15–24 [13]. The treatment with ATRA/cytarabine was repeated with 12 weeks intervals. Treatment with valproic acid started with an intravenous loading dose and thereafter an intravenous infusion for 24 h before the treatment was continued as oral administration guided by the serum level. Samples were collected before treatment (day 1) (Study registration: ClinicalTrials.gov no. NCT00995332 and EudraCT no. 2007–2007–001995-36).

#### Response criteria

The international working group in AML [23, 24] defined complete remission (CR) of AML as (i) less than 5% blast in the bone marrow, no Auer rods and no persistence of extra-medullary disease, and (ii) neutrophil counts above 1.0 × 10^9^/L, platelet levels above 100 × 10^9^/L and erythrocyte transfusion independence. There is no requirement in terms of duration of this response. The MDS response criteria [24, 25] generally require a duration of 8 weeks for the response. The requirements for CR in MDS are (i) less than 5% blasts in the bone marrow and no dysplasia, (ii) hemoglobin level > 11 g/100 ml, neutrophils counts >1.5 × 10^9^/L, platelets counts >100 × 10^9^/L and (iii) no circulating blasts. The MDS criteria also define stable disease as no evidence of progression for at least 8 weeks. Patients referred to as responders in our present study corresponded to patients achieving either (i) complete remission as defined by the AML criteria lasting for at least 8 weeks or (ii) fulfilling the MDS criteria for at least stable disease or hematological improvement with increased normal peripheral blood cell counts.

### Cell preparation

The 28 patients included in the microarray studies represent the subset of patients with high enough peripheral blood blast counts to allow sampling from the peripheral blood of sufficient cells for microarray studies. Leukemic peripheral blood mononuclear cells (PBMCs) were isolated by density gradient separation (Ficoll-Hypaque; NycoMed, Oslo, Norway; specific density 1.077) from peripheral blood of patients with at least 80% of the leukocytes being AML cells. Cells were stored frozen in liquid nitrogen. The percentage of AML blasts among leukemia PBMC exceeded 95% [26].

### Mutation profiling

Submicroscopic mutation profiling of 54 genes frequently mutated in myeloid leukemias was done by the Illuminas TruSight Myeloid Gene Panel and sequenced using the MiSeq system and reagent kit v3 (all from Illumina, San Diego, CA, USA) (Additional file 1: Table S4). Amplicon sequencing library was prepared from 50 ng DNA according to the manufacturer’s instructions with the exception of normalization being done manually. 8–16 samples were sequenced each time and the total DNA input on the flow cell was 15 picomolar. Secondary analysis was performed using MiSeqReporter version 2.4.60.8 (Illumina) mapping to the human genome reference hg19. Sequence alignment of selected variants was manually examined with the Integrative Genomics Viewer (IGV) [27]. Annotation was done by snpSIFT og snpEFF v 4.1. As no matching normal DNA was available variants with >1% minor allele frequency in the 1000 genomes data were presumed to be germline and removed from further interpretations. Synonymous substitutions, intronic variants not in the splice site and variant interpreted as benign or most likely benign are not included. The variant allele frequency (VAF) was calculated for each mutation as number of variant reads divided by total reads. Cut-off for reported variants for VAF was 8% and read depth 100. Only variants interpreted as pathogenic, probably pathogenic and variants of unknown significance are reported. The nomenclature is according to Human Genome Structural Variation consortium.

Fragment analysis of *FLT3* exon 14–15 and *NPM1* exon 12 were done as described in [28] and *CEBPA* mutation analysis as described previously [28].

### RNA preparation, labelling and microarray hybridization

All microarray experiments were performed using the Illumina iScan Reader, which is based upon fluorescence detection of biotin-labelled cRNA. Three hundred ng of total RNA from each sample was reversely transcribed, amplified and Biotin-16-UTP-labelled using the Illumina TotalPrep RNA Amplification Kit (Applied Biosystems/Ambion, USA). The amount and quality of the Biotin-labelled cRNA was controlled both by the NanoDrop spectrophotometer and Agilent 2100 Bioanalyzer. Biotin- labelled cRNA (750 ng) was hybridized to the HumanHT-12 V4 Expression BeadChip according to the manufacturer’s instructions. The HumanHT-12 V4 BeadChip targets 47,231 probes that are mainly derived from genes in the NCBI RefSeq database (Release 38).

### Preprocessing, normalization and annotations of microarray data

Data from the array scanning were investigated in GenomeStudio and J-Express 2012 for quality control measures [29]. All arrays within each experiment were quantile normalized to be comparable before being compiled into an expression profile data matrix. The probe with the highest fluorescence was used in the analyses if the expression of the same gene was examined by different probes. In all our analyses of gene expression profiles we used significant analyses of microarray (SAM) [30], and gene set enrichment analysis (GSEA) [31], to compare different patient subsets or samples. The genes encoding proteins with a known function were classified by using the PANTHER (protein annotation through evolutionary relationship) classification system [32].

## Results

### Classification of patients as responders and non-responders to ATRA/valproic acid

All patients in the present study were included in two previous clinical studies. These studies included 60 patients (20 responders); the characteristics of all patients are summarized in Additional file 1: Table S1 and the characteristics of individual patients included in the present study are presented in Additional file 1: Table S2. Additional analysis of the mutational profiles was possible for 12 responders and 29 non-responders to the treatment (Additional file 1: Table S3; patients 1–12 and 15–43). The effects of antileukemic treatment on gene expression profile were analyzed for eight patients from the study by Ryningen et al. [13]. A high frequency of patients with high-risk disease according to conventional prognostic criteria (i.e. AML relapse, secondary AML, high-risk cytogenetic abnormalities) was seen both for the whole group of 60 patients, the 41 patients included in the study of mutational profiles (Additional file 1: Table S2; patients 7, 8, 17, 23, 26, 31, 38 and 42). None of the patients had low-risk cytogenetic abnormalities.

### Responsiveness to AML-stabilizing therapy was not significantly associated with karyotype or the mutational profile

**Fig. 1:**
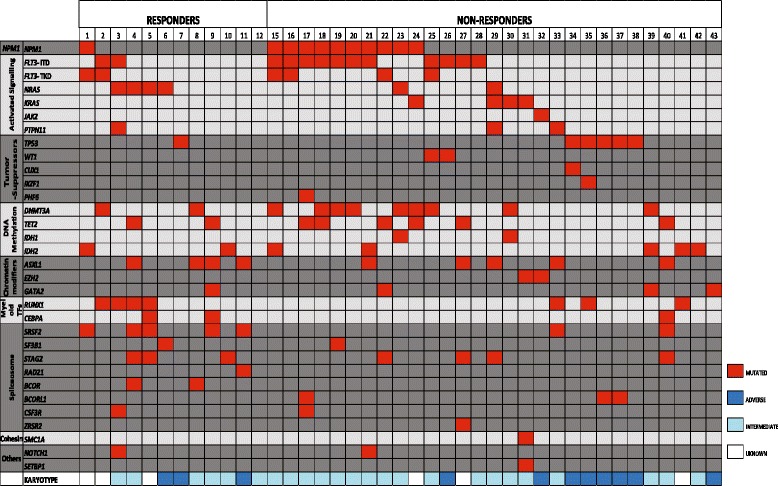
Mutational profiling of responders and non-responders to AML-stabilizing treatment based on ATRA plus valproic acid. Primary AML cells derived from 41 patients (Additional file 1: Table S2, patients 1–2 and 15–43) were analyzed for AML-associated mutations (see Additional file 1: Table S4). The 41 patients included 12 responders and 29 non-responders to the treatment. The patient numbers at the top of the figure refer to the numbers given in Additional file 1: Table S2, and the figure presents the results only for those mutations that were detected for at least one of these patients. The classification of the mutations can be seen in the left part of the figure. The karyotype classification is given at the bottom of the figure, whereas more detailed information about the cytogenetic abnormalities are included in Additional file 1: Table S2

A detailed submicroscopic mutational profile was examined for 41 patients (Additional file 1: Table S2; 12 responders and 29 non-responders); the profile included genes frequently mutated in myeloid malignancies (Additional file 1: Table S4, Fig. 1). Thirty-two of these genes were mutated in at least one of the patients, and according to previous studies [33, 34] these mutations were classified as (i) *NPM1* mutations, (ii) mutations causing activation of intracellular signaling; (iii) mutated tumor suppressor genes; (iv) mutations in genes involved in DNA methylation or (v) chromatin modification; (vi) mutations in genes encoding myeloid transcription factors; (vii) mutated genes important for the spliceosome or (viii) encoding cohesion protein; and (ix) others.

The number of detected mutations differed between patients, the median number being three mutations (range 0–7). Responders and non-responders did not differ with regard to the number of mutations. *FLT3* mutations were most frequent (15 out of 41 patients, 37%) followed by *NPM1* mutations (14/41, 34%). Most patients had at least one mutation causing activation of intracellular signaling (25/41, 61%), and all six patients with *TP53* mutations had an adverse karyotype. Even though *NPM1*, *FLT3, TP53* and *DNMT3A* mutations showed higher frequencies for non-responders than for responders, these differences did not reach statistical significance.

### The gene expression profiles of responders and non-responders to AML-stabilizing treatment differ especially for genes important in nucleic acid binding, intracellular transport, function of hydrolases and modulation of enzyme activity

**Fig. 2:**
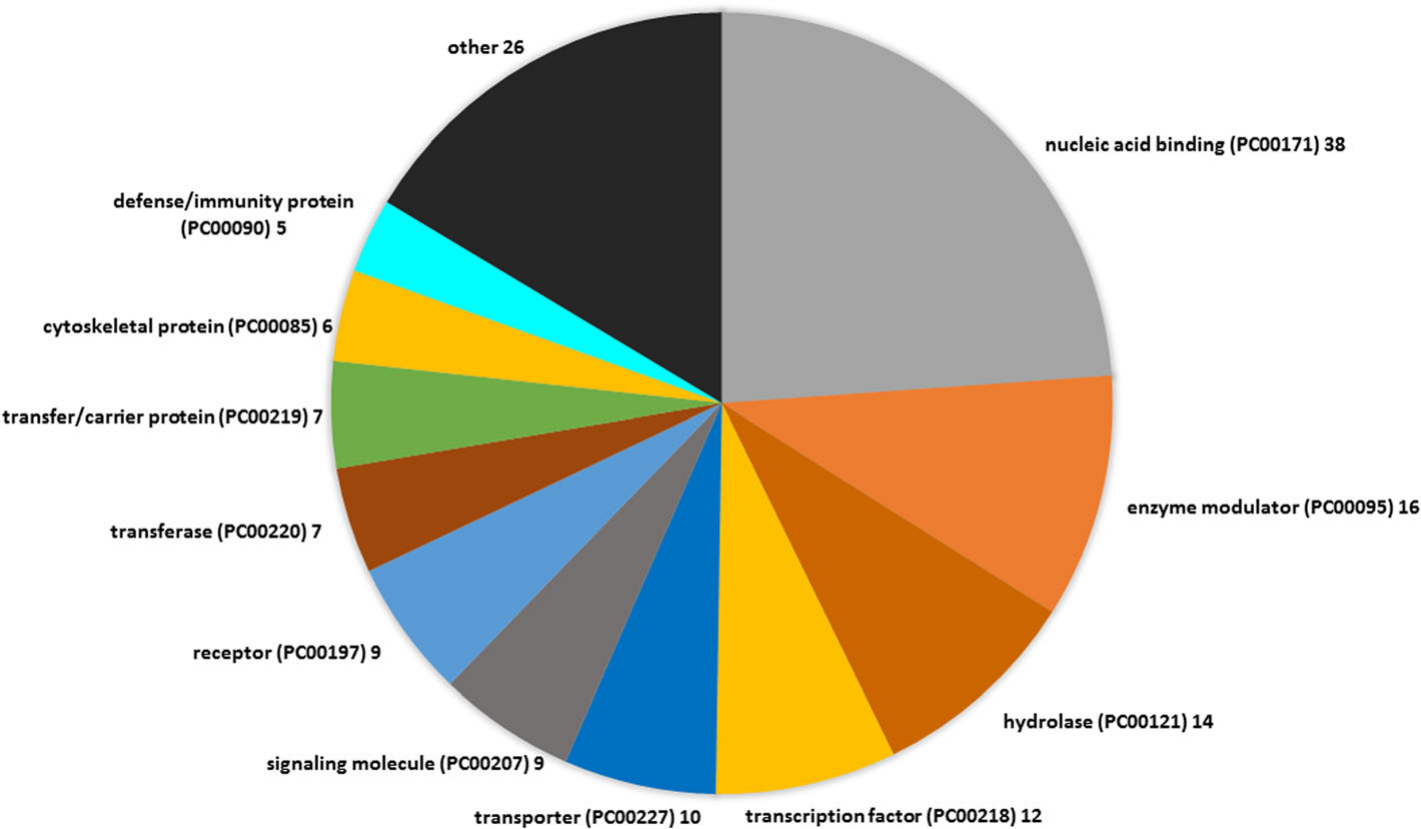
Comparison of the global gene expression profiles for responders and non-responders to the AML-stabilizing treatment based on ATRA and valproic acid – an analysis of the differentially expressed genes based on the function of their encoded proteins. Differentially expressed genes were identified by SAM, and the functional analysis of the encoded proteins was based on the Panther database. Only genes encoding annotated proteins were included in this analysis. The figure thus presents the representative distribution of the genes with known functions that showed differential expression according to the Panther protein class (PS) category. The name of each of the identified classes is given in the figure along with number of genes in each category. Only classes containing ≥ 5 genes are named in the figure. The genes included in each of the five major classes nucleic acid binding, transcription factor, enzyme modulator, hydrolase and receptor are listed in Table 2, and important biological functions of individual genes are described in Additional file 1: Table S6

We compared the global gene expression profiles for pretreatment AML cell samples derived from 28 patients who later could be classified as responders or non-responders to the leukemia-stabilizing treatment. All these AML cell samples were derived from patients with high peripheral blood blast counts, and highly enriched AML cell populations could thereby be prepared by a simple and highly standardized method based on gradient separation. Nineteen of these patients were classified as non-responders and nine as responders according to the criteria previously described in detail by Fredly et al. [13]. We used SAM to compare the global gene expression profiles for responders and non-responders. When setting the d-score to ±2.5 we identified 243 probes that differed significantly between the two groups, and these probes represent 179 different genes (Additional file 1: Table S5). We then used the Panther database to classify the encoded proteins (Fig. 2), and 159 of these genes encoded proteins that could be classified. Genes encoding for proteins that belonged to the “nucleic acid binding” class were overrepresented (38 out of 159 genes), and these genes encoded both DNA binding protein (16 proteins), RNA binding proteins (20 proteins) and nucleases (2 proteins).

All the genes showing at least a 2-fold difference between responders and non-responders are listed in Additional file 1: Table S6. The proteins encoded by these genes included oncogenes (RGL4, LMO4) as well as regulators of protein degradation/activation/modulation (QPCT, ELANE), transcription (HOXA3, HOXA5, PBX3; the only three with decreased expression), iron metabolism (HP, LTF), energy metabolism (MOSC1, CYP4F3, OLR1, SNCA), apoptosis/proliferation (OLFM4, PGLYRP1) and communication (COL17A1, TACSTD2). The Hox genes may be of particular importance, and *HOXA4* expression was also significantly lower although the difference was less than two-fold.

We also performed a GSEA. This alternative analysis also showed that the differentially expressed genes are important for a wide range of cellular function, but several of the identified GO-terms with *p*-value <0.05 describe binding/function/regulation of nucleic acids and showed increased expression for the responders (Table 1).

### The effect of in vivo ATRA therapy on the global gene expression profile of primary human AML cells

Transcriptomic profiling of primary AML cells during in vivo ATRA treatment was only possible for eight patients included in the first study [9]. We first compared AML cell samples derived (i) before start of treatment on day 1, and (ii) after two days of oral ATRA monotherapy (day 3). These eight patients were two responders and six non-responders, and due to this low number of available patients it was not possible to compare the effects of ATRA in responders and non-responders. Thus, by this comparison we identified alterations in global gene expression profiles that are common for responders and non-responders. By this approach we could not identify quantitative differences in the expression of these identified genes and probably not effects of ATRA that are specific for responders/non-responders either. However, differences between responders and non-responders to ATRA may be caused by different downstream responses to common ATRA-induced alterations, and only such mechanisms are likely to be identified by our strategy for analysis.

**Fig. 3:**
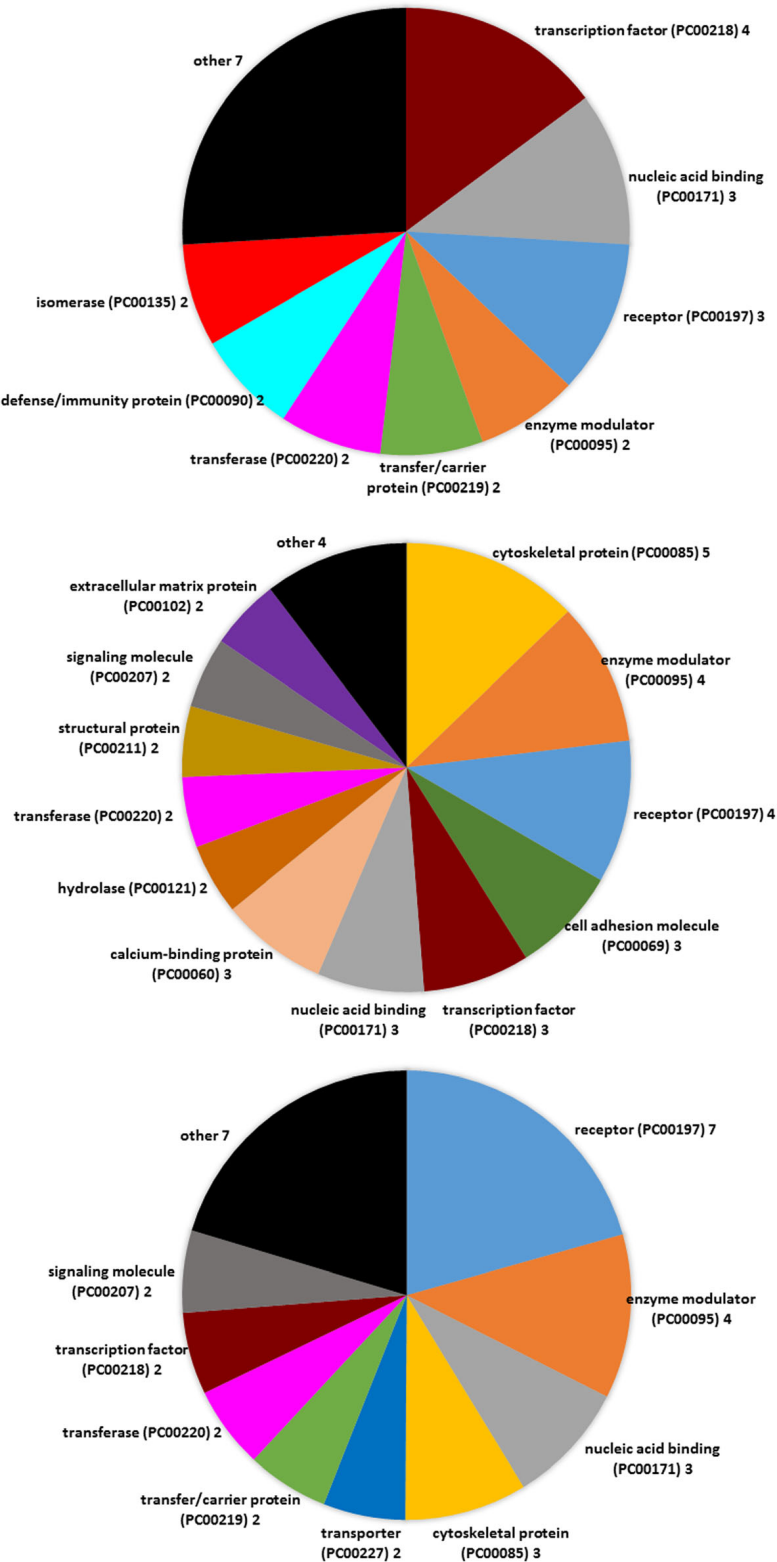
Comparison of the global gene expression profiles – a comparison of primary AML cells sampled before treatment (day 1), after treatment with ATRA alone (day 3) and after triple therapy with ATRA, valproic acid and theophyllamine (day 8). Each part of the figure shows the results for one analysis. The upper part presents the comparison of pretreatment samples and cells collected after 2 days of ATRA therapy (day 3 versus pretreatment samples), the middle figure show the effect of adding valproic acid plus theophyllamine to the ATRA therapy (day 8 versus day 3 samples) and the lower figure shows the effect of the triple combination (pretreatment samples versus AML cells sampled on day 8). The same strategies were used for all three analyses. Differentially expressed genes were first identified by SAM, and the functional analyses of the encoded proteins were based on the Panther database. Only genes encoding annotated proteins were included in these analyses. The figures thus present the representative distribution of the genes with known functions that showed differential expression according to the Panther protein class (PS) category..The name of each of the identified classes is given in the figure along with number of genes in each category. Only classes containing ≥ 2 genes are named. The genes included in each of the five major classes nucleic acid binding, transcription factor, enzyme modulator, hydrolase and receptor are listed in Table 2, and important biological functions of individual genes are described in Additional file 1: Table S6

We performed a SAM analysis that identified the top rankled differently expressed genes with d-score of ±2.0 (400 permutations) when comparing AML cells sampled on day 1 before ATRA treatment and after two days of treatment on day 3. All differentially expressed genes are listed in Additional file 1: Table S5. The Panther classification analysis based on the function of the encoded proteins (Fig. 3); for these analyses we only included the 70 genes (36 upregulated and 34 downregulated on day 3) that were annotated; these genes can be identified from Additional file 1: Table S5). ATRA altered the expression of genes with a wide range of functions, but a major effect of this in vivo therapy was altered expression of genes encoding proteins that show nucleic acid binding and/or being involved in transcriptional regulation. Additional effects were altered expression of receptor-associated genes, whereas the pretreatment differences between responders and non-responders with regard to hydrolases and enzyme modulation (see above) seem to be maintained during ATRA. However, relatively few genes were altered during ATRA therapy for the 5 terms Nucleic acid binding/transcription factors/enzyme modulation/hydrolases/receptors (see Tables 2 and 3), but the genes encode proteins that are involved both in regulation of DNMT3A (*SALL3*) and retinol metabolism (*RBP1*). Finally, there was only a small overlap between those genes showing differential expression when comparing responders and non-responders and those genes being altered by ATRA (Tables 2 and 3). This last observation suggests that the pretreatment differences between responders and non-responders with regard to these 5 terms are maintained during treatment.

**Table 2:** An overview of individual genes that belong to the 5 the GO-terms Nucleic acid binding/transcription factor/hydrolases/enzyme modulation/receptors and their expression in primary human AML cells – differences in gene expression between responders and non-responders

Nucleic acid binding transcription factor			Hydrolases		Enzyme modulation		Receptors	
Increased*	Decreased		Increased	Decreased	Increased	Decreased	Increased	Decreased
*AGAP3*	*AARSD1*	*MBTD1*	*ACP22*	*APOBEC3H*	*AGAP3*	*ARL16*	OLFM4*	*ADRA2C*
*ARAP3*	*APOBEC3H*	*MCART1*	*AFMID*	*ATP2A2*	*ARAP3*	*BIRC3*	*ST7*	HP*
*LMO4*	*ARAP3*	*MRPS15*	LTF*	*CDC14A*	*FBXO8*	*GNB4*	TACSTD2*	*MCHR2*
*NLRX1*	*DDX5I*	*MYCBP2*	*RNASE4*	*ELANE*	*MCF2L2*	HP*		*QRFPR*
*PCBP2*	*CDKN2AIP*	*PBX3*		*GNB4*	*PCBP2*	*MCYBP2*		*THADA*
*RBMI5*	*DDX51*	*PHAX*		HP*	*PI3*	*MYO3B*		*XPO1*
*RNASE4*	*FOXP1*	*SMC4*		*LRAP*	RGL4*	*PHKB*		
*RPL30*	*HIST2H2AC*	*SNRNP70*		*SMC4*	*RNASE4*			
*SNRPB*	HOXA3*	*TDRD1*		*TDP1*	*TP53BP2*			
*TAF8*	*HOXA4*	*THOC*		*XRCC2*				
*UBL4A*	HOXA5*	*XRCC2*						
*WRN*	*LCOR*	*ZMAT3*						
	*LRRF1P1*	*ZNF394*						
	*MBD2*							

The classification of the genes refer to increased/decreased levels in responders compared with non-responders. Only those genes/probes with an annotation were included in the Panther analysis (and thereby also in this table) that is the basis for this classification

Increased or decreased levels means increased expression in responder patients

*Genes with significantly different expression also when comparing responders versus nonresponders (see Additional file 1: Table S5)

**Table 3:** An overview of individual genes that belong to the 5 the GO-terms Nucleic acid binding/transcription factor/hydrolases/enzyme modulation/receptors and their expression in primary human AML cells – effects of in vivo treatment with the triple combination on the gene expression profile

Comparison (Number of genes included in the comparison)	Nucleic acid binding transcription factor		Hydrolases		Enzyme Modulation		Receptors	
	Increased	Decreased	Increased	Decreased	Increased	Decreased	Increased	Decreased
Day 1 versus day 3^a^	*FOXB1* *JRKL*	*SALL3*	*ETHE1*			*RBP1* *KIAA244*		*GRM1* *IGFALS* *PTCHD1*
Day 3 versus day 8^b^	*RSPH9* *SIRT6* *ONECUT*		*SIRT6*	*KLK1*	*ITI4* *ANGBL3*	*KLK1* *GNG12*	*AVPR1B*	*HS.344170* *KLK1* *AVPR1B* *GALR2*
Day 1 versus day 8^c^	*NR2F1* *PRDM13*	*ZBTB7C*			*CCNE2*	*SIPA1L1* *DYNLL2* *ITIH3*	*MRGPRX4* *HCRTR1* *UNC5B* *GPR151*	*GRM1* *AVPR1A* *LRRC55*

These five terms included a major part of the genes that showed differential expression when comparing responders and non-responders. Only genes with known annotations were included in the Panther analysis (and thereby in this table) that forms the basis for the table. The table presents those genes belonging to these five terms altered during treatment. The terms Increased/decreased expression means that the indicated genes showed increased/decreased expression during the investigated therapeutic intervention, i.e. during ATRA treatment day 3, following addition of valproic acid plus theophyllamine day8 and following triple therapy day 8

^a^Increased or decreased on day 3 after ATRA therapy

^b^Increased or decreased on day 8 after addition of valproic acid plus theophyllamine

^c^Increased or decreased on day 8 after triple therapy

### The effect of in vivo treatment with valproic acid plus theophyllamine on the global gene expression profile of primary human AML cells

We compared the gene expression profiles for AML cells derived from the same 8 patients before addition of valproic acid and theophyllamine to the ATRA therapy (days 3) and during treatment with the triple combination (day 8). We then did a SAM analyses identifying the top ranked differently expressed genes with d-score of ±2.0 (400 permutations), and thereafter a Panther protein classification only based on those 81 genes (42 upregulated and 39 downregulated on day 8) that were annotated. The identity of these genes can be seen from Additional file 1: Table S5. The showed that addition of valproic acid/theophyllamine altered the expression of genes that are involved in a wide range of cellular functions, again including altered transcriptional regulation (Fig. 3, Table 3, Additional file 1: Table S7). Altered expression after addition of valproic acid/theophyllamine was observed for relatively few genes, but including genes encoding proteins important for epigenetic regulation (*SIRT6*) and for the kallikrein system (*ITIH4, KLK*) (Additional file 1: Table S7). Very few of these genes differed significantly when comparing pretreatment levels for responders and non-responders (see Tables 2 and 3), i.e. this is similar to the ATRA treatment and suggests that the pretreatment differences between these two patient subsets are maintained during this treatment.

### The overall effect of in vivo treatment with ATRA, valproic acid and theophyllamine on the global gene expression profile of primary human AML cells

We finally compared the effects of the triple drug combination by comparing the global gene expression profiles for primary AML cells derived from the 8 patients before start of treatment and after triple therapy on day 8. The therapeutic serum level for theophyllamine was 55–110 μmol/L. The daily valproic acid dose was increased to the maximal tolerated dose. The therapeutic serum level of valproic acid was 300–600 μmol/L, but the mean valproic acid level during the 5 days of triple treatment varied for individual patients between 178 and 717 μmol/L (median value 407 μmol/L) and did not reach the lower therapeutic limit for 2 of the patients.

We did a SAM analysis identifying the top ranked differentially expressed genes with d-score of ±2.0 (400 permutations), and thereafter we did a Panther protein classification only based on the 76 annotated genes (39 upregulated and 37 downregulated in day 8 samples). The genes included in the terms transcriptional regulation/nucleic acid binding represent only a minority among the genes with altered expression during the triple drug therapy, and the same was true for the term hydrolases (Figure 3, Table 3, Additional file 1: Table S7). Thus, a major part of the pre-therapy differences between responders and non-responders seem to be maintained during the triple treatment and this was also seen for the separate analyses of ATRA and valproic acid/theophyllamine treatment (see above). The triple therapy altered the expression of genes included in several annotations, but major effects seem to be altered receptor expression/function (especially G-protein coupled receptors for neuromediators, i.e. AVPR1B, GALR2, HCRTR1, GPR151) together with altered expression of transcriptional regulators (Additional file 1: Table S7). Finally, altered expression after valproic acid/theophyllaminee was observed for relatively few genes (Table 2) and very few of these genes differed significantly when comparing pre-treatment levels for responders and non-responders (Additional file 1: Table S5), i.e. pre-treatment differences are maintained during treatment.

## Discussion

Most AML patients are elderly and many of these elderly patients as well as younger unfit patients will not benefit from intensive chemotherapy because remission induction is less likely [3, 4] and/or (ii) they have a high risk of severe treatment-related complications and early death due to age, comorbidity or poor performance status [14, 17, 35]. Treatment based on ATRA plus the HDAC inhibitor valproic acid may be an alternative for such patients. However, the in vivo effects of this treatment on the leukemic cells are largely unknown [36, 37].

The treatment of elderly and unfit AML patients often needs to be individualized, and this was also true for the patients included in our present study [9, 13]. Even though our patients were treated according to two different protocols, they all received similar AML-stabilizing treatment (Additional file 1: Table S3) based on ATRA, valproic acid and low-toxicity chemotherapy. Patients with high peripheral blood blast counts at the time of diagnosis received chemotherapy from the start of treatment, otherwise patients in the second protocol received chemotherapy from day 14 and patients in the first protocol received chemotherapy if the peripheral blood blast count increased during treatment. Finally, patients in the first study received theophyllaminee, but this was probably less important with regard to clinical efficiency because the frequency of responders in this study was similar to other previous studies of ATRA + valproic acid alone [14].

ATRA was given at the same daily dose as used in APL therapy and in previous studies of non-APL variants of AML treated with ATRA + valproic acid [9, 14, 17]. The tolerated dose of valproic acid varied between patients [9, 13], but previous studies have demonstrated that clinically relevant effects with improvement of platelet counts can be observed even for patients having concentrations below the therapeutic serum level [14]. Our patients should be regarded as representative for elderly/unfit patients with regard to systemic valproic acid levels [9, 14, 17].

The responses to ATRA + valproic acid based treatment are usually detected after 2–3 weeks [13, 14]. On the other hand, many patients (especially elderly patients) have a short expected survival [13, 14], and if they do not respond to the first AML-stabilizing treatment there may not be sufficient time left to try an alternative treatment. Our present results suggest that gene expression profiling can be used for early identification of patients who are likely to respond to treatment based on ATRA + valproic acid, whereas conventional prognostic criteria (relapse versus first diagnosis, karyotype, molecular genetics) could not be used for prediction of treatment responses.

Several randomized studies have failed to show an effect of ATRA on survival for AML patients receiving intensive and potentially curative chemotherapy (for detailed information and additional references see [38, 39], although a recent study suggests that ATRA improves survival for the subset of patients having *NPM1* mutations or having genetic low risk disease [38]. Thus, the effect of ATRA may be observed only for a subset of patients identified by their genetic abnormalities. For this reason we compared the frequencies of various genetic abnormalities for responders and non-responders to our AML-stabilizing treatment, but we could not detect any significant differences between the two groups. This was also true for *DNMT3*, even though the effect of its regulator *SALL3* (see Additional file 1: Table S7) is altered by ATRA. However, these observations have to be interpreted with great care because we investigated only a limited number of molecular abnormalities and compared relatively small groups of patients. Furthermore, our observation that the responders included several patients with high-risk disease according to conventional prognostic criteria also support the conclusion that conventional prognostic parameters (including cytogenetic and molecular-genetic analysis) have a limited value with regard to predicting responsiveness to AML-stabilizing treatment based on ATRA and valproic acid.

Several studies have described effects of ATRA and valproic acid on gene expression in human AML cells [40–44], and we investigated whether the treatment-induced differences in gene expression or differences between our responders and non-responders included genes that had also been identified in these studies (Additional file 1: Table S5). We first compared our results with 241 genes regulated by retinoic acid [40], but only a minority of these genes were altered by ATRA/valproic acid/theophyllamine (*NR2F1, PCDH12, SFTPA1B, RBP1*) or differed significantly between responders and non-responders (*ABCB1, BIRC3, OLR1*). Secondly, Zheng et al. [41] identified 108 ATRA responsive genes in the NB4 AML cell line, but only *CGREF1* was altered during treatment and only *NCOA3* differed significantly between responders and non-responders. Similarly, Park et al. [42] identified 15 genes altered by in vitro exposure of primary AML cells to ATRA; none of them differed between our responders and non-responders or were altered during treatment. Finally, Bullinger et al. [43] analyzed effects of ATRA on the HL60 AML cell line and detected 427 ATRA-responsive genes; none of their 39 genes with FDR < 0.05 were altered during treatment and only two of these genes (*ARAP3, HOXA3*) differed between our responders and non-responders. However, the decreased levels of *HOXA3*, *HOXA4* and *HOXA5* together with their modulator *PBX3* suggest that *HOX* genes are important for the response to treatment.

Rücker et al. investigated the in vivo effect of valproic acid for AML patients receiving intensive induction treatment [44]. Neither the expression of their 50 top-ranked genes, the 20 genes in their valproic acid-associated miRNA profile nor their 9 response-predicting genes were altered during treatment of our patients or showed differential expression in responders/non-responders.

Expression a stem cell-like mRNA signature seems to be associated with an adverse prognosis in AML; this has been shown both for a leukemic stem cell related profile (34 genes), hematopoietic stem cell related profile (32 genes) and recently by using a 17-gene stemness scoring system [45, 46]. However, only two of these genes (*ABCB1* and *HOXA5*) differed between responders and non-responders, and none of the genes were altered during treatment (Additional file 1: Table S4).

Thus, our present results confirm that both ATRA and valproic acid can alter the expression of a large number of genes involved in a wide range of important cellular processes in primary human AML cells, but only a small number of genes previously shown to be responsive to ATRA or valproic acid were associated with response to treatment or were altered during in vivo treatment of our patients. This lack of overlap suggests that the effects of ATRA/valproic acid on gene expression in human AML cells depend on the biological context during drug exposure.

Recent studies have demonstrated that the mRNA expression of the oncogene *EVI1,* that is important in myeloid malignancies, is induced by ATRA and act as a modulator of ATRA responses [47–49].

Furthermore a substantial part of AML patients with enhanced expression of *EVI1* seem to respond to ATRA by induction of differentiation and decreased clonogenic capacity of myeloid blasts [50]. However, *EV11* was not associated with ATRA responsiveness and was not induced during ATRA treatment in our patients. These observations are also consistent with the observation that the effects of ATRA depends on the biological context; the *EVI1* observations described above were based on in vitro studies of various cells lines and these effects of *EVI1*/ATRA may then be different from primary AML cells exposed to ATRA in vivo.

Only eight patients were available for global gene expression analyses during in vivo treatment. When analyzing pre-therapy samples in responders and non-responders we conclude that these two groups differ mainly in their expression of genes included in the terms nucleic acid binding, transcription factors, hydrolase and enzyme modulators. These pre-therapy differences were maintained during the triple treatment both when comparing pre-therapy expression with the expression after ATRA alone (day 3 samples) and triple therapy (day 8 samples). The most important differences observed on day 8 compared with pre-therapy samples was altered expression by several receptors. Thus, the final effect of the triple in vivo treatment is maintenance of pretreatment differences between responders and non-responders and the most striking treatment-induced difference being increased expression of several receptors.

Several receptors were upregulated during treatment, and many of them were G-protein coupled receptors and/or receptors for neuromediators. The functions of these receptors in leukemogenesis are largely unknown, although a previous study also suggested that they are expressed by malignant hematopoietic cells and are then involved in growth regulation [51]. Thus, the altered expression of these receptors may thus contribute to the final effect of our AML-stabilizing treatment.

## Conclusions

A subset of AML patients responds to disease-stabilizing therapy based on ATRA + valproic acid; the responders include several patients with relapsed/chemoresistant disease and patients with high-risk disease based on their genetic abnormalities. We could not detect any significant differences between responders and non-responders when comparing the frequencies of their genetic abnormalities. Responders and non-responders could be identified by differences in their global gene expression profiles, especially differences in the expression of genes encoding proteins that are important for transcriptional regulation. These differences are maintained during treatment; the triple therapy has only minor effects on the expression of transcriptional regulators but they altered the expression of several receptors.

## Additional file 1

Additional file 1: Table S1. The characteristics of the 60 patients included in the two clinical studies. **Table S2.** Clinical and biological characteristics of the patients included in the study. **Table S3.** AML-stabilizing treatment based on ATRA plus valproic acid; a summary and comparison of the two treatment regimen. **Table S4.** Analysis of 54 submikrocopic mutations in primary human AML cells. **Table S5**. Differences in global gene expression profiles by primary human AML cells derived from responders and non-responders to AML-stabilizing treatment. **Table S6**. Differences in global gene expression profiles by primary human AML cells derived during AML-stabilizing treatment. **Table S7.** Differentially expressed genes identified from comparison of primary AML cells before and after treatment. (DOCX 55 kb)

## Abbreviations

AML: Acute myelogenous leukemia
APL: Acute promyelocytic leukemia
ATRA: All-trans retinoic acid
CR: Complete remission
GO: Gene-ontology
GSEA: Gene set enrichment analysis
HDAC: Histone deacetylase
MDS: Myelodysplastic syndrome
PBMC: Peripheral blood mononuclear cells
SAM: Significant analyses of microarray
TGFB: Transforming growth factor beta
VAF: Variant allele frequency
