# Supplementary material for: Disease-stabilizing treatment based on all-trans retinoic acid and valproic acid in acute myeloid leukemia – identification of responders by gene expression profiling of pretreatment leukemic cells
Source: BMC Cancer. 2017 Sep 6;17:630. doi: 10.1186/s12885-017-3620-y (PMC5586053; doi:10.1186/s12885-017-3620-y)
Supplement: Additional file 1: Table S1. — The characteristics of the 60 patients included in the two clinical studies. Table S2. Clinical and biological characteristics of the patients included in the study. Table S3. AML-stabilizing treatment based on ATRA plus valproic acid; a summary and comparison of the two treatment regimen. Table S4. Analysis of 54 submikrocopic mutations in primary human AML cells. Table S5. Differences in global gene expression profiles by primary human AML cells derived from responders and non-responders to AML-stabilizing treatment. Table S6. Differences in global gene expression profiles by primary human AML cells derived during AML-stabilizing treatment. Table S7. Differentially expressed genes identified from comparison of primary AML cells before and after treatment. (DOCX 55 kb) [file 12885_2017_3620_MOESM1_ESM.docx]

**Additional file 1**

**Disease-stabilizing treatment based on all-trans retinoic acid and valproic acid in acute myeloid leukemia – identification of responders by gene expression profiling of pretreatment leukemic cells**

Håkon Reikvam, Randi Hovland, Rakel Brendsdal Forthun, Sigrid Erdal, Bjørn Tore Gjertsen, Hanne Fredly, Øystein Bruserud

**Table S1.** The characteristics of the 60 patients included in the two clinical studies; the data from the two studies are presented separately.

|  | **Ryningen et al.** | **Fredly et al.** |
| --- | --- | --- |
|  |  |  |
| Number of included patients | 24 | 36 |
| Gender (male/female) | 13/11 | 14/22 |
| Age, median and range (years) | 72 (47-86) | 77 (48-90) |
|  |  |  |
| AML relapse | 7 | 8 |
| AML at first time of diagnosis |  |  |
| de novo AML | 8 | 13 |
| AML secondary to MDS | 5 | 11 |
| AML secondary to MPN | 3 | 2 |
| AML secondary to chemotherapy | 1 | 2 |
|  |  |  |
| Karyotype (frequency) |  |  |
| Good | 0 % | 0 % |
| Intermediate | 10 % | 20 % |
| Adverse | 40 % | 30 % |
| Normal | 50 % | 50 % |
|  |  |  |
| CD34 positivity (frequency) | 86 % | 70 % |
|  |  |  |
| Four-weeks mortality | 25% | 33% |
|  |  |  |

MPN, myeloproliferative neoplasia; MDS, myelodysplastic syndrome.

**References to Table S1:**

Ryningen A, Stapnes C, Lassalle P, Corbascio M, Gjertsen BT, Bruserud O. A subset of patients with high-risk acute myelogenous leukemia shows improved peripheral blood cell counts when treated with the combination of valproic acid, theophylline and all-trans retinoic acid. Leuk Res. 2009 Jun;33(6):779-87. doi: 10.1016/j.leukres.2008.10.005. Epub 2008 Nov 12. PubMed PMID: 19007987.

Fredly H, Ersvær E, Kittang AO, Tsykunova G, Gjertsen BT, Bruserud O. The combination of valproic acid, all-trans retinoic acid and low-dose cytarabine as disease-stabilizing treatment in acute myeloid leukemia. Clin Epigenetics. 2013 Aug 1;5(1):13. doi: 10.1186/1868-7083-5-13. PubMed PMID: 23915396; PubMed Central

PMCID: PMC3765924.

**Table S2.** Clinical and biological characteristics of the patients included in the study. The table presents the data for all patients included in the analysis of AML cell mutations, grey shadow over the patient number indicates those patients included in the studies of global gene expression profiles (patients 7, 8, 17, 23, 26, 31, 38 and 42 used in the studies of drug effects; patients 13* and 14* not included in mutation analyses).

|  | **Survival^1^** | **Sex** | **Age^2^** | **AML status** | **FAB** | **CD34^3^** | **CD117^3^** | **Cytogenetics^4^** |
| --- | --- | --- | --- | --- | --- | --- | --- | --- |
| **RESPONDERS TO TREATMENT** | | | | | | | | |
| 1 | 55 | M | 71-80 | de novo | M1 | Nt | Nt | Nt |
| 2 | 19 | F | 71-80 | Seconday (MDS) | M2 | Nt | Pos | t(1;5); t(2;3) |
| 3 | 56 | M | 71-80 | Secondary (MDS) | M1 | Pos | Nt | Multiple |
| 4 | 20 | F | ≥81 | Seconday (MDS) | M1 | Pos | Pos | Normal |
| 5 | 60 | F | 71-80 | de novo | M1 | Neg | Neg | Normal |
| 6 | 40 | F | 61-70 | Relapse | M4 | Pos | Pos | Multiple |
| 7 | 22 | M | ≥81 | de novo | M1 | Pos | Pos | Multiple |
| 8 | 14 | F | 61-70 | Relapse | M1 | Pos | Pos | Normal |
| 9 | 21 | F | ≥81 | Secondary (MDS) | M1 | Pos | Pos | Normal |
| 10 | 43 | M | ≤60 | MDS/AML relapse | M1 | Pos | Pos | Normal |
| 11 | 82 | M | ≥81 | Secondary (PV) | M2 | Neg | Neg | Monosomi 7 |
| 12 | 15 | M | ≥81 | Seconday (MDS) | M1 | Pos | Pos | Normal |
| 13* | 5 | M | 71-80 | de novo | M1 | Pos | Pos | Multiple |
| 14* | 7 | M | 71-80 | Secondary (MDS) | M1 | Pos | Pos | Nt |
| **NONRESPONDERS TO THE TREATMENT** | | | | | | | | |
| 15 | 1 | M | ≤60 | Relapse | M5 | Neg | Pos | Normal |
| 16 | 3 | F | 61-70 | Relapse | M2 | Neg | Pos | Normal |
| 17 | 1 | F | ≥81 | de novo | M2 | Neg | Pos | Normal |
| 18 | 2 | F | ≤60 | Relapse | M4 | Neg | Nt | Normal |
| 19 | 5 | F | 71-80 | Seconday (MDS) | M1/2 | Pos | Nt | Normal |
| 20 | 2 | F | 71-80 | de novo | M1 | Neg | Pos | Normal |
| 21 | 4 | F | 71-80 | de novo | M1 | Neg | Pos | Normal |
| 22 | 7 | M | 71-80 | Relapse | M2 | Neg | Pos | Normal |
| 23 | 2 | F | 61-70 | Secondary (chemo) | M4 | Neg | Nt | Normal |
| 24 | 1 | M | 71-80 | Secondary (chemo) | M4/5 | Nt | Nt | Nt |
| 25 | 5 | F | ≥81 | de novo | M4 | Pos | Neg | Normal |
| 26 | 1 | M | ≤60 | Relapse | M4 | Pos | Pos | Normal |
| 27 | 3 | M | 71-80 | Seconday (MDS) | M1 | Pos | Pos | Nt |
| 28 | 3 | M | 61-70 | Relapse | M1 | Pos | Pos | Normal |
| 29 | 7 | F | 71-80 | Relapse | M1 | Pos | Pos | del(12) |
| 30 | 1 | M | ≥81 | de novo | M4 | Neg | Neg | Trisomy 8 |
| 31 | 5 | M | 61-70 | Secondary (MF) | M4 | Pos | Pos | Normal |
| 32 | 2 | F | 71-80 | Secondary (PV) | M4/5 | Nt | Nt | Trisomy 13 |
| 33 | 2 | F | ≥81 | de novo | M1 | Pos | Pos | Normal |
| 34 | 5 | M | 61-70 | Seconday (MDS) | M1 | Pos | Pos | Multiple |
| 35 | 7 | F | 61-70 | de novo | M1 | Pos | Pos | Multiple |
| 36 | 6 | F | ≥81 | Seconday (MDS) | M2 | Pos | Pos | Multiple |
| 37 | 20 | F | 61-70 | Secondary (MDS) | M2 | Neg | Pos | Multiple |
| 38 | 4 | F | ≤60 | Li Fraumeni,  Secondary (chemo) | M0 | Pos | Pos | Multiple |
| 39 | 8 | M | 61-70 | Relapse | M1 | Pos | Nt | Normal |
| 40 | 3 | M | 61-70 | Seconday (MDS) | M1 | Pos | Pos | Normal |
| 41 | 6 | F | ≥81 | de novo | M1 | Pos | Pos | Nt |
| 42 | 13 | M | 71-80 | de novo | M0 | Pos | Pos | Normal |
| 43 | 2 | F | ≥81 | de novo | M0 | Pos | Neg | del5 (q13q33) |
|  | | | | | | | | |

**Footnotes Table S2:**

^1^ Survival presented in weeks.

^2^ Age is given in range in years.

^3^ Positivity defined as at least 20% positively stained cells

Abbreviations: Chemo, previously chemotherapy; F, female; M, male; Nt, not tested; MDS, myelodysplastic syndrome; MF, myelofibrosis; PV, polycythemia vera.

^4^ Multiple means three or more unrelated chromosome abnormalities.

**Table S3.** AML-stabilizing treatment based on ATRA plus valproic acid; a summary and comparison of the two treatment regimen.

|  | **Ryningen et al. (PubMed PMID: 19007987)** | **Fredly et al. (PubMed PMID: 23915396)** |
| --- | --- | --- |
|  |  |  |
| **Duration of each cycle** | 12 weeks | 12 weeks |
| **Duration of the treatment** | Until AML progression with peripheral blood blast counts exceeding 100 x 10^9^/L | Until AML progression with peripheral blood blast counts exceeding 100 x 10^9^/L |
|  |  |  |
| **ATRA treatment** |  |  |
| Start of treatment and duration | Day 1 | Day 7 |
| Duration | 14 days, repeated by 12 weeks intervals | 14 days, repeated by 12 weeks intervals |
| Dose | 22,5 mg/m^2^ twice daily | 22,5 mg/m^2^ twice daily |
|  |  |  |
| **Valproic acid** |  |  |
| Start of treatment | Day 3, continued until progression | Day 1, continued until progression |
| Administration | Initial intravenous treatment, later oral treatment | Initial intravenous treatment, later oral treatment |
| Dosage | The maximal tolerated dose, also guided by the serum levels | The maximal tolerated dose, also guided by the serum levels |
|  |  |  |
| **Theophyllamine** | Initial intravenous infusion later oral treatment guided by the serum level. | Not given |
|  |  |  |
| **Low-dose cytarabine** | Allowed to control hyperleukocytosis if peripheral blood blasts >50 x 10^9^/L; it was then administered as 20 mg/m^2^ once daily for 10 days with 4-8 weeks intervals. | Cytarabine 20 mg/m^2^ subcutaneously (sc) once daily for 10 days (days 15-24) with 12 weeks intervals. |
|  |  |  |
| **Individualized chemotherapy; guidelines for additional treatment** |  |  |
| Indications | Hyperleukocytosis or increasing peripheral blood blast counts during treatment | Hyperleukocytosis or increasing peripheral blood blast counts during treatment |
| Low-dose cytarabine | As described above added to ATRA/theophyllamine/valproic acid if hyperleukocytosis or increased peripheral blood blast counts. | For patients with peripheral blood blast counts exceeding 50 x 10^9^/L at the time of diagnosis cytarabine sc was started at day 1 together with valproic acid (same dosage as described above). The interval of the treatment was decreased to 4-8 weeks if peripheral blood blast counts increased before start of the next 12-weeks cycle. |
| Hydroxyurea and 5-  Mercaptopurine | Oral treatment with hydroxyurea or mercaptopurin was allowed if hyperleukocytosis or increasing peripheral blood blast counts. | Oral treatment with hydroxyurea or mercaptopurin was allowed as an alternative treatment if hyperleukocytosis or increasing peripheral blood blast counts despite low-dose cytarabine. |
|  |  |  |

**Table S4.** Analysis of 54 submikrocopic mutations in primary human AML cells.

| **GENE** | **Target region (exon)** | **Amplicons pr gene** | **Mean reads** |
| --- | --- | --- | --- |
| ABL1 | 4-6 | 4 | 6036 |
| ASXL1 | 12 | 19 | 6920 |
| ATRX | 8-10, 17-31 | 25 | 4744 |
| BCOR | full | 39 | 3757 |
| BCORL1 | full | 36 | 3215 |
| BRAF | 15 | 1 | 7897 |
| CALR | 9 | 1 | 839 |
| CBL | 9 | 4 | 5523 |
| CBLB | 9,10 | 3 | 2971 |
| CBLC | 9,10 | 2 | 4109 |
| CDKN2A | full | 8 | 3420 |
| CEBPA | full | 5 | 1221 |
| CSF3R | 14-17 | 8 | 2080 |
| CUX1 | full | 50 | 3883 |
| DNMT3A | full | 26 | 3964 |
| ETV6 | full | 10 | 5174 |
| EZH2 | full | 21 | 6070 |
| FBXW7 | 9,10,11 | 6 | 5506 |
| Flt3 | ex20 | 1 | 11353 |
| Flt3 | 14-15 | 3 | 10595 |
| GATA1 | 2 | 2 | 3534 |
| GATA2 | 2-6 | 9 | 1161 |
| GNAS | 8 | 2 | 5996 |
| HRAS | 2 | 1 | 1327 |
| HRAS | 3 | 2 | 789 |
| IDH1 | 4 | 2 | 4391 |
| IDH2 | 4 | 1 | 3997 |
| IKZF1 | full | 11 | 4040 |
| JAK2 | 12,17 | 2 | 4589 |
| JAK3 | 13 | 1 | 154 |
| KDM6A | full | 41 | 4267 |
| KIT | 10,13,17 | 8 | 4140 |
| KMT2A | 1,3,5,6,7,8,27 | 7 | 6437 |
| KRAS | 2,3 | 3 | 5896 |
| KRAS |  |  |  |
| MPL-ex10 | 10 | 1 | 3771 |
| MYD88 ex3, 5 | 4 | 4 | 3690 |
| NOTCH1 | 26, 27, 28, 34 | 14 | 2191 |
| NPM1 | 11 | 1 | 3145 |
| NRAS | 2 | 1 | 13048 |
| NRAS | 3 | 1 | 223 |
| PDGFRA | 12,14,18 | 3 | 6914 |
| PHF6 | full | 12 | 3719 |
| PTEN | 5,7 | 4 | 0 |
| PTPN11 | 3,13 | 3 | 8100 |
| RAD21 | full | 17 | 5355 |
| RUNX1 | full | 13 | 4098 |
| SETPB1 | 4(partial) | 1 | 3296 |
| SF3B1 | 13-16 | 5 | 4156 |
| SMC1A | 2,11,16,17 | 4 | 3347 |
| SMC3 | 10,13,19,23,25,28 | 7 | 4269 |
| SRSF2 | 1 | 2 | 2686 |
| STAG2 | full | 40 | 4326 |
| TET2 ex 3-11 | 3-11 | 42 | 8646 |
| TP53 | 2-11 | 11 | 4257 |
| U2AF1 | 2,6 | 2 | 6766 |
| WT1 | 7,9 | 2 | 4910 |
| ZRSR2 | full | 15 | 3126 |

**Table S5**. Differences in global gene expression profiles by primary human AML cells derived from responders and non-responders to AML-stabilizing treatment. The table gives a summary of those genes that showed differential expression and a fold change of >2.0. The arrows in the left column indicate whether the expression levels were increased (↑) or decreased (↓) in the responder to the triple treatment. The information about each of the genes is based on the Gene database and selected references from the PubMed database.

| **GENE**  **FOLD CHANGE** | **DESCRIPTION** |
| --- | --- |
| ↑MOSC1  2.066 | *Mitochondrial amidoxime reducing component 1.* The mitochondrial amidoxime reducing component mARC is capable of reducing N-oxygenated structures and requires cytochrome b5 and cytochrome b5 reductase for electron transfer. The protein is possibly involved in lipogenesis/lipid metabolism. (**Plitzko B**). |
| ↑HP  2.137 | *Haptoglobin.* Haptoglobin functions to bind free plasma hemoglobin and is thereby involved in iron metabolism. This gene has also been linked to metabolic and autoimmune disorders. |
| ↑RGL4  2.150 | *Ral guanine nucleotide dissociation stimulator like 4.* Increased expression of this oncogene causes increased expression of this gene leads to translocation of the encoded protein to the cell membrane. The encoded protein can activate several pathways, including the Ras-Raf-MEK-ERK cascade. |
| ↑COL17A1  2.192 | *Collagen type XVII alpha 1 chain.* The alpha chain of type XVII collagen is a transmembrane protein and component of hemidesmosomes, multiprotein complexes at the dermal-epidermal basement membrane zone. |
| ↑CYP4F3  2.212 | *Cytochrome P450 family 4 subfamily F member 3.* CYP4F3 encodes a member of the cytochrome P450 superfamily that localize to the endoplasmatic reticulum; these enzymes catalyze many reactions involved in drug metabolism and synthesis of cholesterol, steroids and other lipids (e.g. inactivating and degrading leukotriene B4) |
| ↑LMO4  2.237 | *LIM domain only 4.* The encoded cysteine-rich protein may play a role as a transcriptional regulator or as an oncogene. |
| ↑OLR1  2.399 | *Oxidized low density lipoprotein receptor 1.* This low density lipoprotein receptor belongs to the C-type lectin superfamily and is regulated through the cyclic AMP signaling pathway. The protein may also be involved in the regulation of Fas-induced apoptosis and may play a role as a scavenger receptor. |
| ↑TACSTD2  2.413 | *Tumor associated calcium signal transducer 2.* The gene encodes a carcinoma-associated cell surface receptor that transduces calcium signals. |
| ↑HEMGN  2.624 | *Hemogen.* The gene encodes a nuclear protein that is important for expansion of hematopoiesis, and its expression is regulated by GATA1 and HOXB4 (**Jiang, Yang, Yang**) |
| ↑QPCT  2.703 | *Glutaminyl-peptide cyclotransferase.* This gene encodes glutaminyl cyclase that is responsible for the presence of pyroglutamyl residues in various peptides. |
| ↑SNCA  2.750 | *Synuclein alpha.* Alpha-synuclein is a member of the synuclein family; these proteins inhibit phospholipase D2 selectively. |
| ↑LTF  2.962 | *Lactoferrin.* This gene is a member of the transferrin family and its protein is an iron-binding protein. The protein is involved in regulation of iron homeostasis, growth, differentiation and protection against cancer development. |
| ↑OLFM4  3.270 | *Olfactomedin 4.* This gene was originally cloned from human myeloblasts. The encoded protein is an antiapoptotic factor that promotes tumor growth and is an extracellular matrix glycoprotein that facilitates cell adhesion. |
| ↑BPI  3.280 | *Bactericidal/permeability-increasing protein.* This gene encodes a lipopolysaccharide binding protein. It is associated with human neutrophil granules. |
| ↑PGLYRP1  3.627 | peptidoglycan recognition protein 1. The protein complexes with HSP70 and then seems to have a proapoptotic effect (**Sashchenko**). |
| ↑TCN1  3.645 | *Transcobalamin 1.* This gene encodes a vitamin B12-binding protein and facilitates the transport of cobalamin into cells. |
| ↑ELANE  4.173 | *Elastase, neutrophil expressed.* Elastases are serine proteases that hydrolyze many proteins; it functions as a regulator of hematopoiesis. |
|  |  |
| ↓HOXA3  -2.111 | *Homeobox A3.* The gene encodes a DNA-binding transcription factor. |
| ↓PBX3  -2.424 | *PBX homeobox 3.* The encoded protein cooperates with HOXA and MEIS1 in leukemogenesis and its expression is possibly associated with an adverse prognosis in AML (**Guo, Garcia-Cuellar, Dickson, Li, Li**). |
| ↓HOXA5  -3.862 | *Homeobox A5.* This gene encodes a DNA-binding transcription factor, among its effects is upregulation of the tumor suppressor p53. |

**References to Table S5:**

Dickson GJ, Liberante FG, Kettyle LM, O'Hagan KA, Finnegan DP, Bullinger L, Geerts D, McMullin MF, Lappin TR, Mills KI, Thompson A. HOXA/PBX3 knockdown impairs growth and sensitizes cytogenetically normal acute myeloid leukemia cells to chemotherapy. Haematologica. 2013 Aug;98(8):1216-25. doi:

10.3324/haematol.2012.079012. Epub 2013 Mar 28. PubMed PMID: 23539541; PubMed

Central PMCID: PMC3729901.

Garcia-Cuellar MP, Steger J, Füller E, Hetzner K, Slany RK. Pbx3 and Meis1 cooperate through multiple mechanisms to support Hox-induced murine leukemia. Haematologica. 2015 Jul;100(7):905-13. doi: 10.3324/haematol.2015.124032. Epub 2015 Apr 24. PubMed PMID: 25911551; PubMed Central PMCID: PMC4486225.

Guo H, Chu Y, Wang L, Chen X, Chen Y, Cheng H, Zhang L, Zhou Y, Yang FC, Cheng T, Xu M, Zhang X, Zhou J, Yuan W. PBX3 is essential for leukemia stem cell maintenance in MLL-rearranged leukemia. Int J Cancer. 2017 Jul 15;141(2):324-335. doi: 10.1002/ijc.30739. Epub 2017 May 8. PubMed PMID: 28411381.

Jiang J, Yu H, Shou Y, Neale G, Zhou S, Lu T, Sorrentino BP. Hemgn is a direct transcriptional target of HOXB4 and induces expansion of murine myeloid progenitor cells. Blood. 2010 Aug 5;116(5):711-9. doi:

10.1182/blood-2009-07-235341. Epub 2010 Apr 14. PubMed PMID: 20393131; PubMed Central PMCID: PMC2918328.

Li Z, Zhang Z, Li Y, Arnovitz S, Chen P, Huang H, Jiang X, Hong GM, Kunjamma RB, Ren H, He C, Wang CZ, Elkahloun AG, Valk PJ, Döhner K, Neilly MB, Bullinger L, Delwel R, Löwenberg B, Liu PP, Morgan R, Rowley JD, Yuan CS, Chen J. PBX3 is an important cofactor of HOXA9 in leukemogenesis. Blood. 2013 Feb

21;121(8):1422-31. doi: 10.1182/blood-2012-07-442004. Epub 2012 Dec 20. PubMed PMID: 23264595; PubMed Central PMCID: PMC3578957.

Li Z, Huang H, Li Y, Jiang X, Chen P, Arnovitz S, Radmacher MD, Maharry K, Elkahloun A, Yang X, He C, He M, Zhang Z, Dohner K, Neilly MB, Price C, Lussier YA, Zhang Y, Larson RA, Le Beau MM, Caligiuri MA, Bullinger L, Valk PJ, Delwel R, Lowenberg B, Liu PP, Marcucci G, Bloomfield CD, Rowley JD, Chen J. Up-regulation of a HOXA-PBX3 homeobox-gene signature following down-regulation of miR-181 is

associated with adverse prognosis in patients with cytogenetically abnormal AML. Blood. 2012 Mar 8;119(10):2314-24. doi: 10.1182/blood-2011-10-386235. Epub 2012 Jan 17. PubMed PMID: 22251480; PubMed Central PMCID: PMC3311258.

Plitzko B, Ott G, Reichmann D, Henderson CJ, Wolf CR, Mendel R, Bittner F, Clement B, Havemeyer A. The involvement of mitochondrial amidoxime reducing components 1 and 2 and mitochondrial cytochrome b5 in N-reductive metabolism in human cells. J Biol Chem. 2013 Jul 12;288(28):20228-37. doi: 10.1074/jbc.M113.474916. Epub 2013 May 23. PubMed PMID: 23703616; PubMed Central PMCID: PMC3711290.

Sashchenko LP, Dukhanina EA, Yashin DV, Shatalov YV, Romanova EA, Korobko EV, Demin AV, Lukyanova TI, Kabanova OD, Khaidukov SV, Kiselev SL, Gabibov AG, Gnuchev NV, Georgiev GP. Peptidoglycan recognition protein tag7 forms a cytotoxic complex with heat shock protein 70 in solution and in lymphocytes. J Biol Chem. 2004 Jan 16;279(3):2117-24. Epub 2003 Oct 29. PubMed PMID: 14585845.

Yang LV, Wan J, Ge Y, Fu Z, Kim SY, Fujiwara Y, Taub JW, Matherly LH, Eliason J, Li L. The GATA site-dependent hemogen promoter is transcriptionally regulated by GATA1 in hematopoietic and leukemia cells. Leukemia. 2006 Mar;20(3):417-25. PubMed PMID: 16437149.

Yang LV, Nicholson RH, Kaplan J, Galy A, Li L. Hemogen is a novel nuclear factor specifically expressed in mouse hematopoietic development and its human homologue EDAG maps to chromosome 9q22, a region containing breakpoints of hematological neoplasms. Mech Dev. 2001 Jun;104(1-2):105-11. PubMed PMID: 11404085.

**Table S6**. Differences in global gene expression profiles by primary human AML cells derived during AML-stabilizing treatment. The table gives a summary of differentially expressed genes belonging to the terms nucleic acid binding/transcription factor/hydrolase/enzyme modifiers/receptors and showing a significant alteration during treatment. We compared the expression levels (i) during ATRA treatment, i.e. day 3 samples during ATRA therapy versus pretreatment samples on day 1 (During ATRA); (ii) before and during addition of valproic acid plus theophyllamin, i.e. day 8 samples during treatment versus day 3 samples during treatment with ATRA alone (Before and after valproic acid plus theophyllamine); and (iii) the effect of the triple combination, i.e. samples collected during triple therapy on day 8 versus pretreatment samples collected on day 1 (Triple therapy). The arrows in the left column indicate whether the expression levels were increased (↑) or decreased (↓) in samples collected during the indicated treatment, i.e. the effects of ATRA alone, valproic acid plus theophyllamine when added to ATRA, and the triple combination, respectively. The information is based on the Gene database and selected references from the PubMed database.

| **Nucleic acid binding-transcription factor** | |
| --- | --- |
| **During ATRA** |  |
| ↑FOXB1 | *Forkhead box B1.* Transcription factor. |
| ↑JRKL | *JRK-like.* The protein may act as a nuclear regulatory protein. |
| ↓SALL3 | *Spalt like transcription factor 3.* This gene encodes a zinc-finger protein. This protein binds to DNA methyltransferase 3 alpha (DNMT3A), and reduces DNMT3A-mediated CpG island methylation. Silencing of this gene with altered DNA methylation may have a role in leukemogenesis. |
| ↓RSPH9 | *Radial spoke head 9 homolog.* The methylation pattern of this gene can be altered in human cancer and may have a prognostic impact (**Yamada, Yoon**). |
| **Before and after valproic acid plus theophyllamine** | |
| ↑SIRT6 | *Sirtuin 6.* The encoded member of the sirtuin family of NAD-dependent enzymes is localized to the nucleus, exhibits ADP-ribosyl transferase and histone deacetylase activities, and plays a role in DNA repair, maintenance of telomeric chromatin, lipid and glucose metabolism. |
| ↑ONECUT1 | *One cut homeobox 1.* The encoded member of the Cut homeobox family of transcription factors may influence a variety of cellular processes including glucose metabolism and cell cycle regulation. It may also be associated with cancer. |
| **Triple therapy** | |
| ↑NR2F1 | *Nuclear receptor subfamily 2 group F member 1.* The protein is a nuclear hormone receptor and transcriptional regulator. |
| ↑PRDM13 | *PR/SET domain 13.* Transcription factor; its methylation pattern may be altered in certain human malignancies (**Rubicz**). |
| ↓ZBTB7C | *Zinc finger and BTB domain containing 7C.* The encoded protein is regarded as a proto-oncoprotein. It acts as a transcriptional regulator, regulates cancer cell proliferation and interacts both with Fas and p53 (**Hur, Jeon, Jeon, Jeon, Lee**). |
| **Hydrolases** | |
| **During ATRA** | |
| ↑ETHE1 | *ETHE1, persulfide dioxygenase.* This gene encodes a member of the metallo beta-lactamase family of iron-containing proteins involved in the mitochondrial sulfide oxidation pathway. |
| **Before and after valproic acid plus theophyllamine** | |
| ↑SIRT6 | See above, nucleic acid binding/transcription factor. |
| ↓KLK1 | *Kalikrein 1.* Kallikreins are a subgroup of serine proteases having diverse physiological functions. Growing evidence suggests that many kallikreins are implicated in carcinogenesis and some have potential as novel cancer and other disease biomarkers. |
| **Enzyme modulation** | |
| **During ATRA** | |
| ↓RBP1 | *Retinol binding protein 1.* The encoded protein is involved in the transport of retinol (vitamin A alcohol) to peripheral tissue. |
| ↓KIAA1244 | *ARFGEF family member 3.* Guanine nucleotide exchange factor. |
| **Before and after valproic acid plus theophyllamine** | |
| ↑ITIH4 | *Inter-alpha-trypsin inhibitor heavy chain family member 4.* The encoded extracellular protein is cleaved by plasma kallikrein. |
| ↓KLK1 | Kalikrein. See above, hydrolases. |
| ↓GNG12 | *G protein subunit gamma 12.*A long non-coding RNA, possibly involved in regulation of expression of certain G-proteins (Niemczyk)? |
| ↑AGBL3 | *ATP/GTP binding protein like 3.* |
| **Triple therapy** | |
| ↓SIPA1L1 | *Signal-induced proliferation-associated 1 like 1.* Involved in regulation of endocytosis (**Wang**) |
| ↓DYNLL1 | *Dynein light chain LC8-type 1.* Cytoplasmic dyneins are large enzyme complexes that are involved in intracellular transport and motility. |
| ↑CCNE2 | *Cyclin E2.* The encoded protein forms a complex with and functions as a regulatory subunit of cyclin dependent kinase 2 (CDK2). It specifically interacts with CIP/KIP family of CDK inhibitors, and plays a role in cell cycle G1/S transition. |
| ↓ITIH3 | *Inter-alpha-trypsin inhibitor heavy chain 3.* This gene encodes the heavy chain subunit of the pre-alpha-trypsin inhibitor complex that seems to stabilize extracellular matrix through its ability to bind hyaluronic acid. |
| **Receptors** | |
| **During ATRA** | |
| ↓GRM1 | *Glutamate metabotropic receptor 1.* This protein is a metabotropic glutamate receptor that functions by activating phospholipase C and whose activity is mediated by a G-protein-coupled phosphatidylinositol-calcium second messenger system. Increased signaling may be important in human cancers. |
| ↓IGFALS | *Insulin like growth factor binding protein acid labile subunit.* The encoded serum protein binds insulin-like growth factors, increasing their half-life and their vascular localization. |
| ↓PTCHD1 | *Patched domain containing 1.* This membrane protein that may be implicated in hedgehog signaling. |
| **Before and after valproic acid plus theophyllamine** | |
| ↓ ELFN2 | *Extracellular leucine rich repeat and fibronectin type III domain containing 2.* |
| ↓KLK1 | See above, hydrolases |
| ↑AVPR1B | *Arginine vasopressin receptor 1B.* The encoded protein acts as receptor for arginine vasopressin, belongs to the subfamily of G-protein coupled receptors and stimulates a phosphatidylinositol-calcium second messenger system. It shows high expression in certain human tumors. |
| ↓GALR2 | *Galanin receptor 2.* Galanin is a 30-amino acid non-C-terminally amidated peptide. The actions of galanin are mediated through specific G protein-coupled receptors. The primary signaling mechanism for this receptor is through the phospholipase C/protein kinase C pathway and it couples efficiently to both the Gq and Gi proteins to simultaneously activate 2 independent signal transduction pathways. |
| **Triple therapy** | |
| ↑MRGPRX4 | *MAS related GPR family member X4.* Possibly involved in carcinogenesis (Gylfe)? |
| ↑HCRTR1 | *Hypocretin receptor 1.* The encoded G-protein coupled receptor binds orexin A and orexin B. The receptor is also expressed by leukemic hematopoietic stem cells (Kronenwett). |
| ↓AVPR1A | *Arginine vasopressin receptor 1A.* The encoded receptor for arginine vasopressin belongs to the G-protein coupled receptor family. Its activity is mediated by G proteins which stimulate a phosphatidylinositol-calcium second messenger system and it mediates cell contraction, proliferation and glycogenolysis. |
| ↑UNC5B | *Unc-5 netrin receptor B.* This member of the netrin family of receptors, belonging to the group of socalled dependence receptors (DpRs) that are involved in pro- and anti-apoptotic processes and possibly also carcinogenesis. |
| ↓LRRC55 | *Leucine rich repeat containing 55.* The protein may be a involved in potassium channel functions (Li). |
| ↑GPR151 | *G protein-coupled receptor 151.* The encoded protein is similar to the galanin receptor subfamily of G protein-coupled receptors. |

**References to Table S6:**

Gylfe AE, Kondelin J, Turunen M, Ristolainen H, Katainen R, Pitkänen E, Kaasinen E, Rantanen V, Tanskanen T, Varjosalo M, Lehtonen H, Palin K, Taipale M, Taipale J, Renkonen-Sinisalo L, Järvinen H, Böhm J, Mecklin JP, Ristimäki A, Kilpivaara O, Tuupanen S, Karhu A, Vahteristo P, Aaltonen LA. Identification of candidate oncogenes in human colorectal cancers with microsatellite instability. Gastroenterology. 2013 Sep;145(3):540-3.e22. doi: 10.1053/j.gastro.2013.05.015. Epub 2013 May 16. PubMed PMID: 23684749.

Hur MW, Yoon JH, Kim MY, Ko H, Jeon BN. Kr-POK (ZBTB7c) regulates cancer cell proliferation through glutamine metabolism. Biochim Biophys Acta. 2017 May 30;1860(8):829-838. doi: 10.1016/j.bbagrm.2017.05.005. [Epub ahead of print] PubMed PMID: 28571744.

Jeon BN, Yoon JH, Kim MK, Choi WI, Koh DI, Hur B, Kim K, Kim KS, Hur MW. Zbtb7c is a molecular 'off' and 'on' switch of Mmp gene transcription. Biochim Biophys Acta. 2016 Nov;1859(11):1429-1439. doi: 10.1016/j.bbagrm.2016.09.004. Epub 2016 Sep 17. PubMed PMID: 27646874.

Jeon BN, Kim YS, Choi WI, Koh DI, Kim MK, Yoon JH, Kim MY, Hur B, Paik PD, Hur MW. Kr-pok increases FASN expression by modulating the DNA binding of SREBP-1c and Sp1 at the proximal promoter. J Lipid Res. 2012 Apr;53(4):755-66. doi: 10.1194/jlr.M022178. Epub 2012 Feb 13. PubMed PMID: 22331133; PubMed Central PMCID: PMC3307652.

Jeon BN, Kim MK, Choi WI, Koh DI, Hong SY, Kim KS, Kim M, Yun CO, Yoon J, Choi KY, Lee KR, Nephew KP, Hur MW. KR-POK interacts with p53 and represses its ability to activate transcription of p21WAF1/CDKN1A. Cancer Res. 2012 Mar 1;72(5):1137-48. doi: 10.1158/0008-5472.CAN-11-2433. Epub 2012 Jan 17. PubMed PMID: 22253232.

Kronenwett R, Butterweck U, Steidl U, Kliszewski S, Neumann F, Bork S, Blanco ED, Roes N, Gräf T, Brors B, Eils R, Maercker C, Kobbe G, Gattermann N, Haas R. Distinct molecular phenotype of malignant CD34(+) hematopoietic stem and progenitor cells in chronic myelogenous leukemia. Oncogene. 2005 Aug 11;24(34):5313-24. PubMed PMID: 15806158.

Lee KM, Choi WI, Koh DI, Kim YJ, Jeon BN, Yoon JH, Lee CE, Kim SH, Oh J, Hur MW. The proto-oncoprotein KR-POK represses transcriptional activation of CDKN1A by MIZ-1 through competitive binding. Oncogene. 2012 Mar 15;31(11):1442-58. doi: 10.1038/onc.2011.331. Epub 2011 Aug 1. PubMed PMID: 21804610.

Li Q, Fan F, Kwak HR, Yan J. Molecular basis for differential modulation of BK channel voltage-dependent gating by auxiliary γ subunits. J Gen Physiol. 2015 Jun;145(6):543-54. doi: 10.1085/jgp.201511356. PubMed PMID: 26009545; PubMed Central PMCID: PMC4442785.

Niemczyk M, Ito Y, Huddleston J, Git A, Abu-Amero S, Caldas C, Moore GE, Stojic L, Murrell A. Imprinted chromatin around DIRAS3 regulates alternative splicing of GNG12-AS1, a long noncoding RNA. Am J Hum Genet. 2013 Aug 8;93(2):224-35. doi: 10.1016/j.ajhg.2013.06.010. Epub 2013 Jul 18. PubMed PMID: 23871723; PubMed Central PMCID: PMC3738830.

Rubicz R, Zhao S, Geybels M, Wright JL, Kolb S, Klotzle B, Bibikova M, Troyer D, Lance R, Ostrander EA, Feng Z, Fan JB, Stanford JL. DNA methylation profiles in African American prostate cancer patients in relation to disease progression. Genomics. 2016 Feb 21. pii: S0888-7543(16)30012-X. doi: 10.1016/j.ygeno.2016.02.004. [Epub ahead of print] PubMed PMID: 26902887; PubMed Central PMCID: PMC4992660.

Wang PJ, Lin ST, Liu SH, Kuo KT, Hsu CH, Knepper MA, Yu MJ. Vasopressin-induced serine 269 phosphorylation reduces Sipa1l1 (signal-induced proliferation-associated 1 like 1)-mediated aquaporin-2 endocytosis. J Biol Chem. 2017 May 12;292(19):7984-7993. doi: 10.1074/jbc.M117.779611. Epub 2017 Mar 23.

PubMed PMID: 28336531; PubMed Central PMCID: PMC5427275.

Yamada N, Yasui K, Dohi O, Gen Y, Tomie A, Kitaichi T, Iwai N, Mitsuyoshi H, Sumida Y, Moriguchi M, Yamaguchi K, Nishikawa T, Umemura A, Naito Y, Tanaka S, Arii S, Itoh Y. Genome-wide DNA methylation analysis in hepatocellular carcinoma. Oncol Rep. 2016 Apr;35(4):2228-36. doi: 10.3892/or.2016.4619. Epub 2016 Feb 11. PubMed PMID: 26883180.

Yoon HY, Kim YJ, Kim JS, Kim YW, Kang HW, Kim WT, Yun SJ, Ryu KH, Lee SC, Kim WJ. RSPH9 methylation pattern as a prognostic indicator in patients with non-muscle invasive bladder cancer. Oncol Rep. 2016 Feb;35(2):1195-203. doi: 10.3892/or.2015.4409. Epub 2015 Nov 11. PubMed PMID: 26575865.

**Table S7.** Differentially expressed genes identified from comparison of primary AML cells before and after ATRA (left), before and after the triple combination ATRA + Valproic acid + theophyllamine (A+V+T, middle left), before and after addition of valproic acid + theophyllamine to the ATRA therapy (V+T, middle right) and leukemic cells derived from responders and non-responders to ATRA + valproic acid + theophyllamine (right). The profiles were compared with the results from five previous studies describing genes that can be altered by exposure of AML cells to retinoids, ATRA or valproic acid. Overlapping genes between our present study and the previous studies are identified by the different colours.

|  |  |  |  | |  |
| --- | --- | --- | --- | --- | --- |
| RETINOID: Balmer JE, Blomhoff R, J Lipid Res 2002, 43, 1773-1808 | | | | |  |
| ATRA: Bullinger L et al. Clin Cancer Res 2013, 19, 2562-2571. | | | | |  |
| ATRA: Zheng P-Z et al, Proc Natl Acad Sci (USA) 2005, 102, 7653-7658. | | | | | |
| VALPROIC ACID: Rücker FG et al., Epigenetics 2016, 11, 517-525 | | | | |  |
| STEM CELL: Eppert K et al, Nat Med 22011, 17, 1086-1093 | | | | |  |
|  |  |  |  | |  |
| **ATRA** | **A+V+T** | **V+T** | **Responder versus non-responder** | | |
| BMP5 | AQP8 | ABCB11 | AARSD1 | |  |
| C1ORF161 | ATG5 | AFP | ABCB1 | |  |
| C3ORF65 | AVPR1A | AGBL3 | ACOT2 | | LRAP |
| CA7 | BOK | AVPR1B | ACPL2 |  | LRRFIP1 |
| CCDC27 | C12ORF69 | C1QL4 | ADRA2C | | MAGT1 |
| CRYGN | C17ORF72 | C4ORF40 | AFMID | | MAP2K3 |
| DEFB103A | C7ORF40 | C8ORF46 | AGAP3 | | MAP2K3 |
| DLEU2L | CCNE2 | CALML3 | APOBEC3H | | MAPK14 |
| ETHE1 | CFHR1 | CGREF1 | ARAP3 | | MAPK14 |
| FAM135B | DGKI | DCP1B | ARL16 | | MBD2 |
| FBXO40 | DYNLL2 | DIS3L2 | ARRDC4 | | MBTD1 |
| FLJ45455 | FAM122C | DNCL2A | ARRDC4 | | MCART1 |
| FOXB1 | FBXO40 | EME2 | ATP2A2 | | MCF2L2 |
| GAFA3 | FLJ35282 | FBXL15 | BIRC3 | | MCHR2 |
| GRM1 | FLJ41327 | FLJ46363 | BLZF1 | | MDGA1 |
| HS.127352 | GPR151 | GALR2 | BMS1P5 | | MED28 |
| HS.139204 | GRM1 | GNG12 | BPI | | MGC40489 |
| HS.161330 | HCRTR1 | GPR153 | C12orf34 | | MOSC1 |
| HS.164254 | HS.131656 | HCG4P6 | C14orf153 | | MRFAP1L1 |
| HS.209165 | HS.170701 | HISPPD1 | C14orf85 | | MRPL39 |
| HS.25131 | HS.25131 | HS.148257 | C15orf63 | | MRPL52 |
| HS.344169 | HS.34747 | HS.176498 | C1orf210 | | MRPS15 |
| HS.385784 | HS.396327 | HS.195045 | C20orf27 | | MYCBP2 |
| HS.443993 | HS.436627 | HS.245405 | C20orf55 | | MYCT1 |
| HS.482589 | HS.486010 | HS.344170 | C21orf24 | | MYO3B |
| HS.538103 | HS.545338 | HS.354827 | C22orf13 | | NCOA3 |
| HS.539083 | HS.545544 | HS.374023 | C2orf55 | | NDUFV2 |
| HS.545544 | HS.552008 | HS.381884 | C3orf34 | | NDUFV3 |
| HS.549914 | HS.560741 | HS.538226 | C7orf49 | | NLRX1 |
| HS.563166 | HS.561057 | HS.538683 | C9orf156 | | NME3 |
| HS.565461 | HS.563550 | HS.545670 | CDC14A | | NUP188 |
| HS.565863 | HS.575816 | HS.546003 | CDKN2AIPNL | | OLFM4 |
| HS.566481 | HS.577755 | HS.549460 | CHRNA5 | | OLR1 |
| HS.575608 | HS.582446 | HS.549914 | CLCA1 | | OR2G3 |
| HS.575816 | HS.583755 | HS.550285 | CLEC4D | | PATE2 |
| HS.580199 | HSPB6 | HS.561575 | CLEC5A | | PAWR |
| HS.91147 | IER2 | HS.565086 | COL17A1 | | PBX3 |
| IGFALS | INDO | HS.574008 | CSF3R | | PCBP2 |
| ITGA8 | ITIH3 | HS.575241 | CYLN2 | | PGLYRP1 |
| JRKL | KIAA1660 | HS.575608 | CYP4F3 | | PHAX |
| KIAA1244 | KRTAP12-3 | IDO1 | DDX51 | | PHKB |
| MAGEB5 | LRRC55 | IL1F6 | DEF8 | | PI3 |
| MAPK15 | MGC15885 | ITIH4 | DEM1 | | PIAS2 |
| MIR1908 | MIR210 | JAKMIP3 | DKFZP586I1420 | | PLS3 |
| MIR224 | MIR382 | KIAA0953 | DNASE1L3 | | PPID |
| MIR708 | MIR708 | KIF18B | EID2B | | PRIM2 |
| NAT8L | MRGPRX4 | KLK1 | ELANE | | QPCT |
| NFKBIL2 | NKG7 | KRT3 | FAM110A | | QRFPR |
| NHLRC1 | NR2F1 | KRT33B | FAM115A | | RBKS |
| OBP2A | OBP2B | LRRC37A3 | FAM119A | | RBM15 |
| OBP2B | OR12D2 | MAGEB6 | FAM122B | | RGL4 |
| OR10G2 | PCDH12 | MIR148A | FAM164A | | RNASE4 |
| OR13C8 | PRDM13 | MIR149 | FAM175A | | RPL30 |
| OR2C1 | PRLH | MIR1914 | FAM35A | | RSPH10B |
| OR4K17 | PRNT | MYL2 | FAM63A | | SDHAF2 |
| OR8B12 | PRR20B | NAT8L | FAM72B | | SEH1L |
| P2RX6P | RFPL3 | ONECUT1 | FAR2 | | SEMA3E |
| PID1 | RIMBP3C | OR4F16 | FBN2 | | SHROOM4 |
| PRUNE2 | SCARNA27 | PAPPA | FBXO8 | | SLC20A1 |
| PSPHL | SEMG2 | PCDHA11 | FCAR | | SLC26A8 |
| PTCHD1 | SFTPA1B | PSMA8 | FCGR3A | | SMC4 |
| RBP1 | SIPA1L1 | RAET1L | FKBP14 | | SMC4 |
| RIBC1 | SNORA50 | RSPH9 | FKBP1P1 | | SMC6 |
| SALL3 | SNORA67 | SCARNA14 | FLJ10916 | | SNCA |
| SCARNA1 | SNORA70 | SIRT6 | FLJ36131 | | SNCA |
| SDC4P | SNORD10 | SNORA59B | FLJ44342 | | SNORD59B |
| SNORD73A | SNORD53 | SNORA68 | FOXP1 | | SNRNP70 |
| SPANXF1 | SPANXF1 | SNORD59B | FUT6 | | SNRPB |
| TMEM232 | TMEM16C | TAF7L | GBAS | | SNX1 |
| UBE2K | TRMT61A | TAS2R50 | GNB4 | | SPIN2B |
|  | TTLL13 | TMCO5A | GRIK2 | | SPRYD3 |
|  | TTR | TMEM16C | GRIPAP1 | | ST7 |
|  | UNC5B | TTTY3 | HARBI1 | | ST7 |
|  | WDR74 | UTP23 | HCG2P7 | | STOM |
|  | ZBTB7C | VRK2 | HEATR6 | | SUV420H1 |
|  | ZNF132 | VWC2 | HEMGN | | TACSTD2 |
|  |  | VWDE | HEMGN | | TAF8 |
|  |  | YKT6 | HIATL2 | | TCN1 |
|  |  | ZNF132 | HIST2H2AC | | TDP1 |
|  |  | ZNF354A | HNRNPU | | TDRD1 |
|  |  |  | HOXA3 | | TDRD1 |
|  |  |  | HOXA4 | | THADA |
|  |  |  | HOXA5 | | THOC1 |
|  |  |  | HP | | TMEM17 |
|  |  |  | HSD17B11 | | TNFSF15 |
|  |  |  | HSPC268 | | TP53BP2 |
|  |  |  | IQCA1 | | TPRG1L |
|  |  |  | KCNH6 | | TRIM13 |
|  |  |  | KIAA1430 | | TRIM54 |
|  |  |  | KIAA1751 | | UBL4A |
|  |  |  | KIAA1967 | | VTRNA1-1 |
|  |  |  | KILLIN | | WRN |
|  |  |  | KPNA5 | | XKR6 |
|  |  |  | KRBA1 | | XPO1 |
|  |  |  | LCOR | | XRCC2 |
|  |  |  | LMO4 | | ZMAT3 |
|  |  |  | LMO4 | | ZNF14 |
|  |  |  | LMOD3 | | ZNF394 |
|  |  |  |  | | ZNF483 |
|  |  |  |  | | ZNF549 |
|  |  |  |  | | ZNF562 |
|  |  |  |  | | ZNF577 |
|  |  |  |  | | ZNF69 |
|  |  |  |  | | ZNF701 |
|  |  |  |  | | ZNF702P |
|  |  |  |  | | ZNF860 |
